# Crystal structure of a new monoclinic polymorph of *N*-(4-methyl­phen­yl)-3-nitro­pyridin-2-amine

**DOI:** 10.1107/S1600536814012227

**Published:** 2014-07-19

**Authors:** Aina Mardia Akhmad Aznan, Zanariah Abdullah, Vannajan Sanghiran Lee, Edward R. T. Tiekink

**Affiliations:** a Department of Chemistry, University of Malaya, 50603 Kuala Lumpur, Malaysia

**Keywords:** crystal structure, amine, pyridine, polymorph, conformation

## Abstract

The title compound, C_12_H_11_N_3_O_2_, is a second monoclinic polymorph (*P*2_1_, with *Z*′ = 4) of the previously reported monoclinic (*P*2_1_/*c*, with *Z*′ = 2) form [Akhmad Aznan *et al.* (2010 ▶). *Acta Cryst.* E**66**, o2400]. Four independent mol­ecules comprise the asymmetric unit, which have the common features of a *syn* disposition of the pyridine N atom and the toluene ring, and an intra­molecular amine–nitro N—H⋯O hydrogen bond. The differences between mol­ecules relate to the dihedral angles between the rings which range from 2.92 (19) to 26.24 (19)°. The geometry-optimized structure [B3LYP level of theory and 6–311 g+(d,p) basis set] has the same features except that the entire mol­ecule is planar. In the crystal, the three-dimensional architecture is consolidated by a combination of C—H⋯O, C—H⋯π, nitro-N—O⋯π and π–π inter­actions [inter-centroid distances = 3.649 (2)–3.916 (2) Å].

## Abstract 2

Molecules in both polymorphs of the title compound display deviations from planarity owing to crystal packing effects.

## Chemical context   

Original inter­est in the mol­ecules related to the title compound revolved around their fluorescence properties (Kawai *et al.*, 2001 ▶; Abdullah, 2005 ▶). The title compound was isolated during an ongoing study of co-crystals formed between carb­oxy­lic acids and pyridine-containing mol­ecules (Arman & Tiekink, 2013 ▶; Arman *et al.*, 2014 ▶), designed to prove the robustness of the {⋯HOC(=O)⋯N(pyridine)} heterosynthon in co-crystals (Shattock *et al.*, 2008 ▶) of functionalized carb­oxy­lic acids with pyridine derivatives. The crystal structure of the title compound has been reported previously as a monoclinic (*P*2_1_/*c*, with *Z*′ = 2) polymorph (Akhmad Aznan *et al.*, 2010 ▶), and the present polymorph was isolated from a failed co-crystallization experiment as detailed in Section 5. The phenomenon of isolating polymorphs from co-crystallization experiments is gaining increasing prominence, especially since the isolation of a second form of aspirin (Vishweshwar *et al.*, 2005 ▶), and led Zaworotko to suggest co-crystallization experiments should also be employed in polymorph screening (Arora & Zaworotko, 2009 ▶).
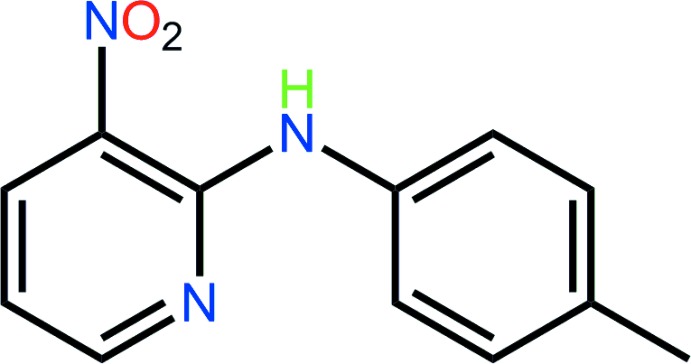


## Structural commentary   

**Figure 1:**
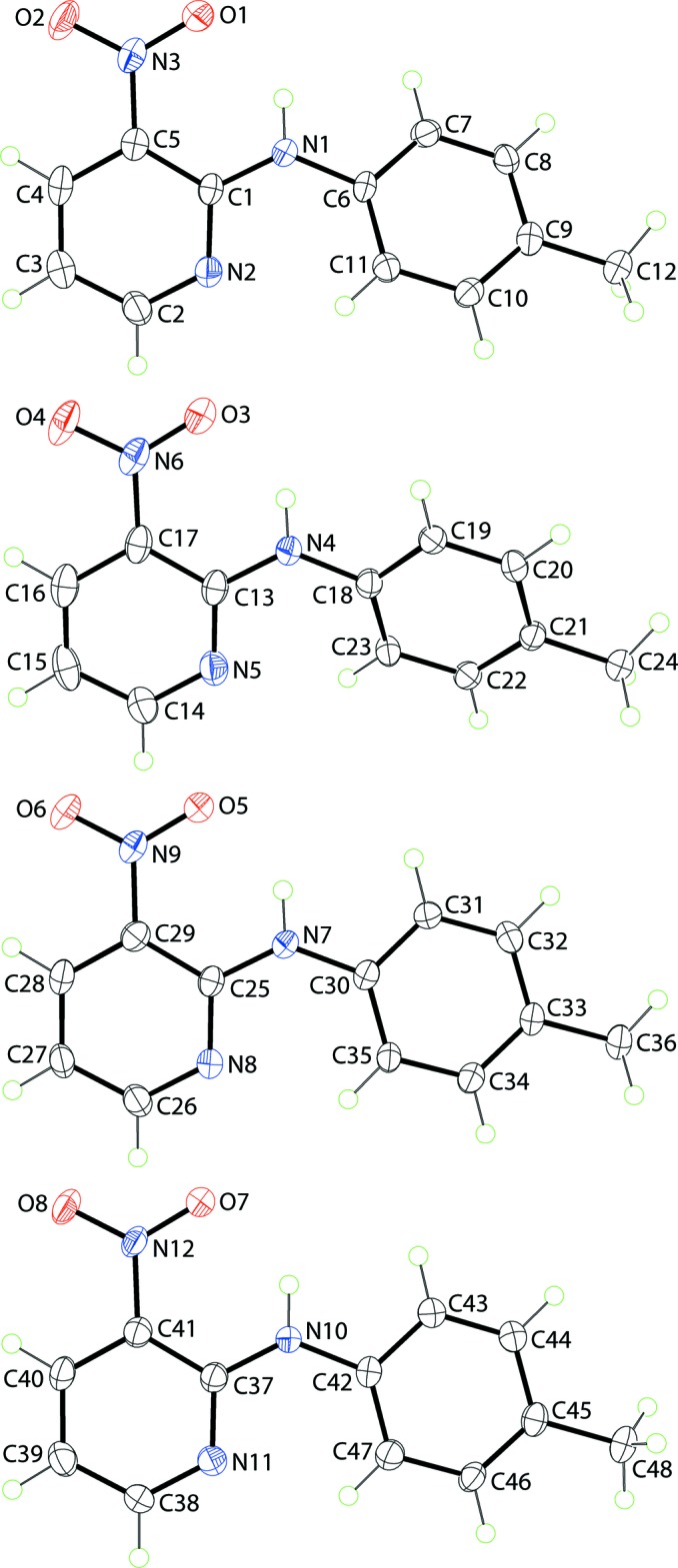
The mol­ecular structures of the four independent mol­ecule in the title compound, showing the atom-labelling scheme and displacement ellipsoids at the 50% probability level.

Four crystallographically independent mol­ecules comprise the asymmetric unit (Fig. 1 ▶). Each mol­ecule features a secondary amine linking nitro­benzene and tolyl groups, with the nitropyridyl N atom *syn* to the toluene ring. An intra­molecular N—H⋯O hydrogen bond closes an *S*(6) loop in each mol­ecule (Table 1 ▶). This feature of the structure confers coplanarity of the nitro group with the pyridyl ring to which it is attached; the maximum deviation from coplanarity is seen in the pyridyl/nitro group dihedral angle of 5.2 (3)°, for the N10-containing mol­ecule. More significant differences are found in the dihedral angles between the two rings, *i.e*. 23.79 (19), 26.24 (19), 6.57 (18) and 2.92 (19)° for the N1-, N4-, N7- and N10-containing mol­ecules, respectively. Similar conformations were observed for the two independent mol­ecules in the previously reported *P*2_1_/*c* polymorph (Aznan Akhmad *et al.*, 2010 ▶). Here, the dihedral angles between the rings were 17.42 (16) and 34.64 (16)°, resembling the N1- and N4-containing mol­ecules in the present study rather than the almost planar N7- and N10-containing mol­ecules.

**Figure 2:**
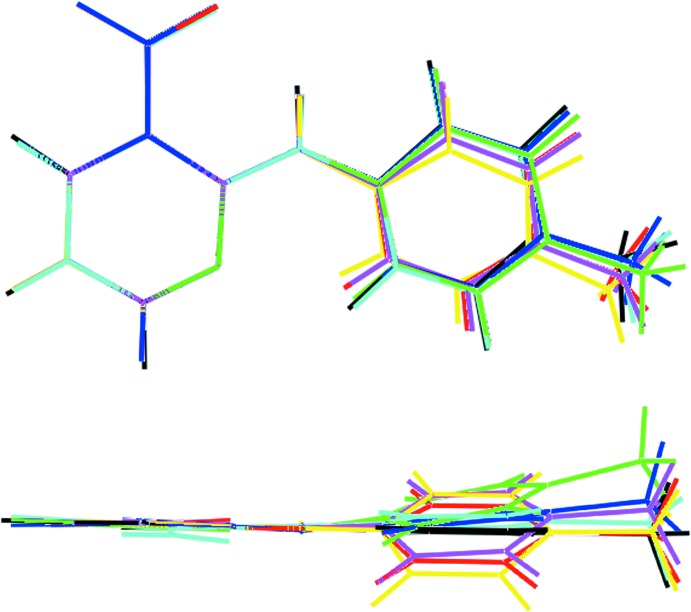
Overlay diagram of conformations of the title compound. The N1-, N4-, N7- and N10-containing mol­ecules determined in the present study are shown in red, pink, blue and aqua, respectively; the N1-, N7- and N10-containing mol­ecules were inverted for a better fit. The green and yellow images correspond to the unique mol­ecules in the known polymorph and the black image corresponds to the geometry-optimized structure.

Geometry optimization calculations were conducted using *GAUSSIAN09* (Frisch *et al.*, 2009 ▶) with the hybrid B3LYP level of theory and the 6-311g+(d,p) basis set. To confirm that a true minimum had been calculated, a frequency calculation was also performed. The gas-phase-optimized structure is strictly planar. An overlay diagram for the six experimentally determined mol­ecules is shown in Fig. 2 ▶ and these are superimposed upon the geometry-optimized structure. Clearly, deviations from planarity in the experimentally determined mol­ecules arise from the dictates of crystal packing.

## Supra­molecular features   

**Figure 3:**
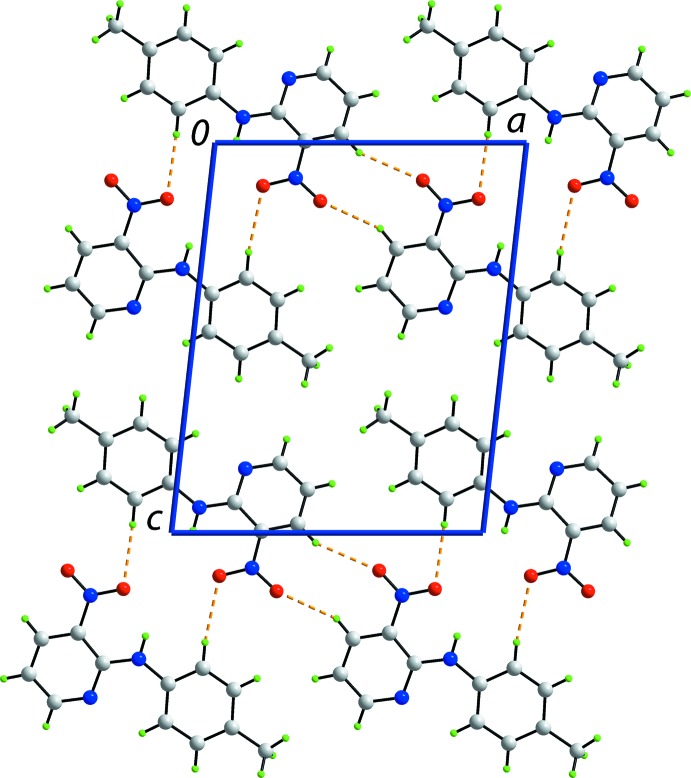
Supra­molecular rows along the *a* axis involving the N1- and N7-containing mol­ecules. The C—H⋯O inter­actions are shown as orange dashed lines.

**Figure 4:**
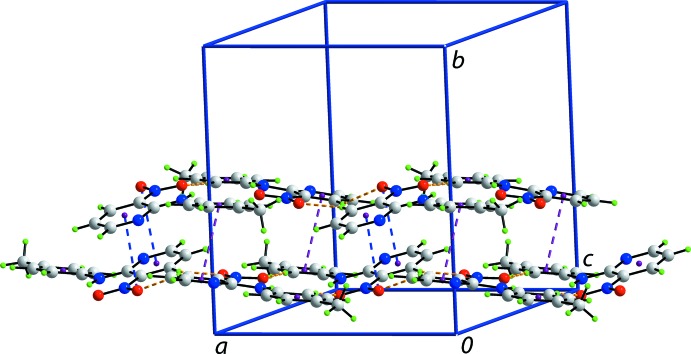
View of the double chain with an undulating topology. The C—H⋯O, N—O⋯π and π–π contacts are shown as orange, blue and purple dashed lines, respectively.

**Figure 5:**
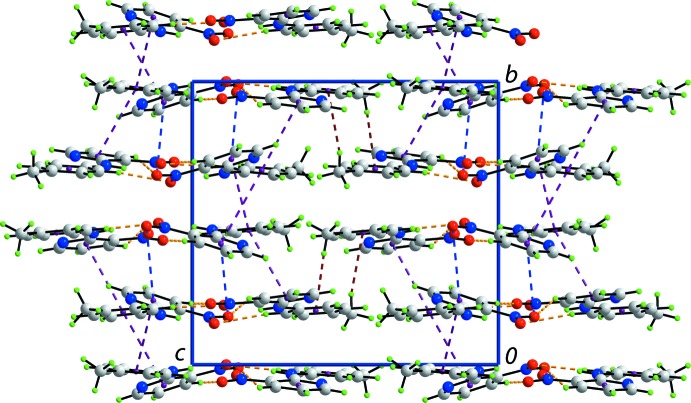
Unit-cell contents shown in projection down the *a* axis. The C—H⋯O, C—H⋯π, N—O⋯π and π–π contacts are shown as orange, brown, blue and purple dashed lines, respectively.

Globally, the crystal packing features alternating layers of mol­ecules that stack along the *b* axis. The first layer comprises N1- and N7-containing mol­ecules that associate *via* C—H⋯O inter­actions (Table 1 ▶). Ten-membered {⋯HC_2_NO}_2_ synthons, with no crystallographically imposed symmetry, are formed *via* pyridine–nitro C—H⋯O inter­actions. Larger, again non-symmetric, 16-membered {⋯HC_2_NC_2_NO}_2_ synthons are formed *via* toluene–nitro C—H⋯O inter­actions (Fig. 3 ▶). These combine to form rows of mol­ecules aligned along the *a* axis. The second independent layer comprises N4- and N10-containing mol­ecules which associate in a similar fashion. However, it is noted that the C40—H40⋯O(nitro) inter­action to close the 10-membered {⋯HC_2_NO}_2_ synthon is a little longer that the standard distance criteria incorporated in *PLATON* (Spek, 2009 ▶). Rows of N1- and N7-containing mol­ecules and rows of N4- and N10-containing mol­ecules are connected into a double chain with an undulating topology *via* π–π and nitro-O⋯π(pyrid­yl) inter­actions (Fig. 4 ▶). The π–π inter­actions occur between toluene C6–C11 and N11-pyridine rings [inter­centroid separation = 3.680 (2) Å; angle of inclination = 4.03 (19)° for symmetry operation (−*x*, *y* − 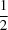, −*z* + 1)], and toluene C18–C23 and N8-pyridine rings [3.649 (2) Å, 3.44 (18)°, −*x* + 1, *y* + 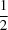, −*z* + 1]. As summarized in Table 1 ▶, the nitro–pyridine O⋯π inter­actions occur between the nitro O2 and O4 atoms and the N5- and N2-containing pyridine rings. Chains are connected into a layer parallel to (010) *via* meth­yl–toluene C—H⋯π inter­actions, and layers are connected into a three-dimensional architecture (Fig. 5 ▶) *via* weaker π–π inter­actions between pyridine and toluene rings: inter­centroid distance for (N4/C1–C5)⋯(C18–C23) = 3.916 (2) Å, with an angle of inclination of 11.04 (19)°, and inter­centroid distance for (C6–C11)⋯(N5/C13–C17) = 3.913 (2) Å, with an angle of inclination of 13.44 (19)° and symmetry operation (*x* − 1, *y*, *z*).

## Database survey   

The most closely related structures in the literature are *N*-(3-chloro­phen­yl)-3-nitro­pyridin-2-amine (Akhmad Aznan *et al.*, 2011 ▶) and 4-[(3-nitro­pyridin-2-yl)amino]­phenol (Cao *et al.*, 2011 ▶). Similar features are evident in these mol­ecules, *i.e*. the intra­molecular N—H⋯O(nitro) hydrogen bond, the coplanarity of the nitro group and pyridine ring, and a conrotatory twist of the two rings, *i.e*. dihedral angles of 9.88 (5) and 84.77 (10)°, respectively. Finally, the structure of the all-phenyl analogue, 2-nitro­diphenyl­amine, has been reported (McWilliam *et al.*, 2001 ▶). Again, the same features are evident and the comparable dihedral angle is 44.45 (7)°.

## Synthesis and crystallization   

*N*-(4-Methyl­phen­yl)-3-nitro­pyridin-2-amine (0.05 g, 0.22 mmol), prepared according to the literature procedure of Akhmad Aznan *et al.* (2010 ▶), was mixed with 3-nitro­benzoic acid (Merck; 0.03 g, 0.22 mmol) in a 1:1 solution of ethanol and ether (10 ml). The solution was refluxed for 4 h at 350 K. The mixture was then left for slow evaporation and red crystals formed after 3–4 d.

## Refinement   

Crystal data, data collection and structure refinement details are summarized in Table 2 ▶. Carbon-bound H atoms were placed in calculated positions (C—H = 0.95 Å) and were included in the refinement in the riding-model approximation, with *U*_iso_(H) set at 1.2*U*_eq_(C). N-bound H atoms were located in a difference Fourier map but were refined with a distance restraint of N—H = 0.88±0.01 Å and with *U*_iso_(H) set at 1.2*U*_eq_(N). In the absence of significant anomalous scattering effects, 4208 Friedel pairs were averaged in the final refinement.

## Supplementary Material

Crystal structure: contains datablock(s) I, global. DOI: 10.1107/S1600536814012227/hb0011sup1.cif

Structure factors: contains datablock(s) I. DOI: 10.1107/S1600536814012227/hb0011Isup2.hkl

Supporting information file. DOI: 10.1107/S1600536814012227/hb0011Isup3.cml

CCDC reference: 1004278

Additional supporting information:  crystallographic information; 3D view; checkCIF report

## Figures and Tables

**Table 1:** Hydrogen-bond geometry (Å, °)

*D*—H⋯*A*	*D*—H	H⋯*A*	*D*⋯*A*	*D*—H⋯*A*
N1—H1*N*⋯O1	0.88 (2)	1.93 (2)	2.632 (4)	135 (3)
N4—H4*N*⋯O3	0.88 (2)	1.92 (3)	2.630 (4)	137 (4)
N7—H7*N*⋯O5	0.88 (2)	1.92 (3)	2.622 (4)	136 (4)
N10—H10*N*⋯O7	0.89 (2)	1.96 (2)	2.636 (4)	132 (3)
C31—H31⋯O1^i^	0.95	2.50	3.444 (4)	173
C28—H28⋯O2^ii^	0.95	2.59	3.414 (4)	145
C4—H4⋯O6^iii^	0.95	2.55	3.440 (4)	157
C7—H7⋯O5^iv^	0.95	2.38	3.331 (4)	174
C43—H43⋯O3^ii^	0.95	2.49	3.436 (4)	172
C40—H40⋯O4^v^	0.95	2.64	3.489 (5)	149
C19—H19⋯O7^iii^	0.95	2.42	3.364 (4)	176
C16—H16⋯O8^vi^	0.95	2.52	3.398 (4)	153
N3—O3⋯*Cg*(N5,C13–C17)^vii^	1.24 (1)	3.55 (1)	3.449 (3)	75 (1)
N6—O4⋯*Cg*(N2,C1–C5)^viii^	1.24 (1)	3.46 (1)	3.469 (3)	80 (1)
C36—H36*C*⋯*Cg*(C42–C47)^ix^	0.98	2.72	3.698 (4)	174
C48—H48*B*⋯*Cg*(C30–C35)^x^	0.98	2.95	3.868 (4)	156

Symmetry codes: (i) 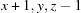; (ii) 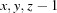; (iii) 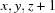; (iv) 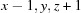; (v) 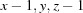; (vi) 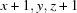; (vii) 
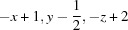
; (viii) 
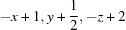
; (ix) 
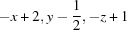
; (x) 
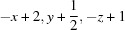
.

**Table 2:** Experimental details

Crystal data	
Chemical formula	C_12_H_11_N_3_O_2_
*M*_r_	229.24
Crystal system, space group	Monoclinic, *P*2_1_
Temperature (K)	100
*a*, *b*, *c* (Å)	11.4079 (6), 13.1968 (8), 14.3681 (7)
β (°)	96.387 (5)
*V* (Å^3^)	2149.7 (2)
*Z*	8
Radiation type	Cu *K*α
μ (mm^−1^)	0.82
Crystal size (mm)	0.20 × 0.20 × 0.04

Data collection	
Diffractometer	Agilent SuperNova Dual with an Atlas detector
Absorption correction	Multi-scan (*CrysAlis PRO*; Agilent, 2013 ▶)
*T*_min_, *T*_max_	0.206, 1.000
No. of measured, independent and observed [*I* > 2σ(*I*)] reflections	24994, 4623, 3633
*R*_int_	0.064
(sin θ/λ)_max_ (Å^−1^)	0.626

Refinement	
*R*[*F* ^2^ > 2σ(*F* ^2^)], *wR*(*F* ^2^), *S*	0.043, 0.124, 1.01
No. of reflections	4623
No. of parameters	629
No. of restraints	5
H-atom treatment	H atoms treated by a mixture of independent and constrained refinement
Δρ_max_, Δρ_min_ (e Å^−3^)	0.27, −0.27

Computer programs: *CrysAlis PRO* (Agilent, 2013 ▶), *SHELXS97* and *SHELXL97* (Sheldrick, 2008 ▶), *ORTEP-3 for Windows* (Farrugia, 2012 ▶), *QMol* (Gans & Shalloway, 2001 ▶) and *DIAMOND* (Brandenburg, 2006 ▶), *publCIF* (Westrip, 2010 ▶).

## supplementary crystallographic information

### Crystal data

**Table:**

C_12_H_11_N_3_O_2_	*F*(000) = 960
*M**_r_* = 229.24	*D*_x_ = 1.417 Mg m^−^^3^
Monoclinic, *P*2_1_	Cu *K*α radiation, λ = 1.54184 Å
Hall symbol: P 2yb	Cell parameters from 4609 reflections
*a* = 11.4079 (6) Å	θ = 3.1–76.4°
*b* = 13.1968 (8) Å	µ = 0.82 mm^−^^1^
*c* = 14.3681 (7) Å	*T* = 100 K
β = 96.387 (5)°	Plate, red
*V* = 2149.7 (2) Å^3^	0.20 × 0.20 × 0.04 mm
*Z* = 8	

### Data collection

**Table:**

Agilent SuperNova Dual diffractometer with an Atlas detector	4623 independent reflections
Radiation source: SuperNova (Cu) X-ray Source	3633 reflections with *I* > 2σ(*I*)
Mirror monochromator	*R*_int_ = 0.064
Detector resolution: 10.4041 pixels mm^-1^	θ_max_ = 75.0°, θ_min_ = 3.1°
ω scan	*h* = −14→14
Absorption correction: multi-scan (*CrysAlis PRO*; Agilent, 2013)	*k* = −16→16
*T*_min_ = 0.206, *T*_max_ = 1.000	*l* = −14→17
24994 measured reflections	

### Refinement

**Table:**

Refinement on *F*^2^	Primary atom site location: structure-invariant direct methods
Least-squares matrix: full	Secondary atom site location: difference Fourier map
*R*[*F*^2^ > 2σ(*F*^2^)] = 0.043	Hydrogen site location: inferred from neighbouring sites
*wR*(*F*^2^) = 0.124	H atoms treated by a mixture of independent and constrained refinement
*S* = 1.01	*w* = 1/[σ^2^(*F*_o_^2^) + (0.0775*P*)^2^ + 0.0456*P*] where *P* = (*F*_o_^2^ + 2*F*_c_^2^)/3
4623 reflections	(Δ/σ)_max_ < 0.001
629 parameters	Δρ_max_ = 0.27 e Å^−^^3^
5 restraints	Δρ_min_ = −0.27 e Å^−^^3^

### Special details

**Table:**

Geometry. All s.u.'s (except the s.u. in the dihedral angle between two l.s. planes) are estimated using the full covariance matrix. The cell s.u.'s are taken into account individually in the estimation of s.u.'s in distances, angles and torsion angles; correlations between s.u.'s in cell parameters are only used when they are defined by crystal symmetry. An approximate (isotropic) treatment of cell s.u.'s is used for estimating s.u.'s involving l.s. planes.
Refinement. Refinement of *F*^2^ against ALL reflections. The weighted *R*-factor *wR* and goodness of fit *S* are based on *F*^2^, conventional *R*-factors *R* are based on *F*, with *F* set to zero for negative *F*^2^. The threshold expression of *F*^2^ > 2σ(*F*^2^) is used only for calculating *R*-factors(gt) *etc*. and is not relevant to the choice of reflections for refinement. *R*-factors based on *F*^2^ are statistically about twice as large as those based on *F*, and *R*- factors based on ALL data will be even larger.

### Fractional atomic coordinates and isotropic or equivalent isotropic displacement parameters (Å^2^)

**Table:**

	*x*	*y*	*z*	*U*_iso_*/*U*_eq_	
O1	0.1693 (2)	0.4996 (2)	1.11071 (17)	0.0305 (6)	
O2	0.3584 (2)	0.4890 (2)	1.14982 (19)	0.0406 (7)	
N1	0.0806 (3)	0.4751 (3)	0.9351 (2)	0.0244 (7)	
H1N	0.069 (3)	0.486 (3)	0.9939 (11)	0.029*	
N2	0.2143 (3)	0.4208 (3)	0.8357 (2)	0.0260 (7)	
N3	0.2712 (3)	0.4837 (2)	1.0903 (2)	0.0278 (6)	
C1	0.1923 (3)	0.4515 (3)	0.9220 (2)	0.0218 (7)	
C2	0.3235 (3)	0.3972 (3)	0.8216 (3)	0.0305 (8)	
H2	0.3364	0.3756	0.7605	0.037*	
C3	0.4211 (3)	0.4013 (3)	0.8889 (3)	0.0321 (8)	
H3	0.4977	0.3834	0.8743	0.039*	
C4	0.4021 (3)	0.4322 (3)	0.9773 (3)	0.0281 (8)	
H4	0.4660	0.4365	1.0256	0.034*	
C5	0.2883 (3)	0.4573 (3)	0.9954 (2)	0.0244 (7)	
C6	−0.0239 (3)	0.4736 (3)	0.8718 (2)	0.0214 (7)	
C7	−0.1293 (3)	0.4664 (3)	0.9128 (2)	0.0250 (7)	
H7	−0.1271	0.4602	0.9789	0.030*	
C8	−0.2366 (3)	0.4684 (3)	0.8576 (2)	0.0245 (7)	
H8	−0.3074	0.4633	0.8864	0.029*	
C9	−0.2432 (3)	0.4777 (3)	0.7603 (3)	0.0242 (7)	
C10	−0.1377 (3)	0.4853 (3)	0.7207 (2)	0.0243 (7)	
H10	−0.1401	0.4916	0.6547	0.029*	
C11	−0.0288 (3)	0.4837 (3)	0.7750 (2)	0.0236 (7)	
H11	0.0419	0.4896	0.7461	0.028*	
C12	−0.3607 (3)	0.4781 (3)	0.7003 (2)	0.0282 (7)	
H12A	−0.3549	0.5194	0.6444	0.042*	
H12B	−0.4211	0.5066	0.7362	0.042*	
H12C	−0.3823	0.4086	0.6816	0.042*	
O3	0.5598 (2)	0.6453 (2)	1.07113 (18)	0.0350 (6)	
O4	0.7456 (3)	0.6690 (2)	1.1166 (2)	0.0416 (7)	
N4	0.4768 (3)	0.6770 (2)	0.8954 (2)	0.0246 (6)	
H4N	0.465 (4)	0.666 (3)	0.9539 (12)	0.030*	
N5	0.6142 (3)	0.7310 (3)	0.7993 (2)	0.0301 (7)	
N6	0.6613 (3)	0.6682 (2)	1.0544 (2)	0.0303 (7)	
C13	0.5895 (3)	0.7002 (3)	0.8844 (3)	0.0252 (8)	
C14	0.7248 (3)	0.7557 (3)	0.7875 (3)	0.0326 (8)	
H14	0.7406	0.7770	0.7270	0.039*	
C15	0.8189 (3)	0.7523 (3)	0.8582 (3)	0.0357 (10)	
H15	0.8962	0.7713	0.8463	0.043*	
C16	0.7964 (3)	0.7208 (3)	0.9452 (3)	0.0327 (9)	
H16	0.8587	0.7161	0.9947	0.039*	
C17	0.6822 (3)	0.6959 (3)	0.9604 (3)	0.0263 (8)	
C18	0.3730 (3)	0.6821 (3)	0.8309 (3)	0.0223 (7)	
C19	0.2680 (3)	0.6955 (3)	0.8700 (2)	0.0229 (7)	
H19	0.2691	0.7027	0.9359	0.027*	
C20	0.1620 (3)	0.6984 (3)	0.8131 (2)	0.0241 (7)	
H20	0.0909	0.7065	0.8409	0.029*	
C21	0.1568 (3)	0.6898 (3)	0.7160 (2)	0.0230 (7)	
C22	0.2628 (3)	0.6757 (3)	0.6785 (2)	0.0237 (7)	
H22	0.2616	0.6689	0.6125	0.028*	
C23	0.3706 (3)	0.6712 (3)	0.7341 (2)	0.0233 (7)	
H23	0.4416	0.6608	0.7065	0.028*	
C24	0.0417 (3)	0.6970 (3)	0.6543 (2)	0.0268 (7)	
H24A	0.0370	0.6424	0.6078	0.040*	
H24B	0.0368	0.7627	0.6223	0.040*	
H24C	−0.0238	0.6906	0.6927	0.040*	
O5	0.8659 (2)	0.4620 (2)	0.14411 (17)	0.0357 (6)	
O6	0.6805 (2)	0.4394 (2)	0.09688 (17)	0.0326 (6)	
N7	0.9406 (3)	0.4660 (2)	0.3234 (2)	0.0250 (6)	
H7N	0.953 (4)	0.478 (3)	0.2651 (12)	0.030*	
N8	0.7985 (2)	0.4312 (2)	0.42218 (19)	0.0242 (6)	
N9	0.7626 (3)	0.4480 (2)	0.1605 (2)	0.0266 (6)	
C25	0.8268 (3)	0.4458 (3)	0.3342 (2)	0.0217 (7)	
C26	0.6856 (3)	0.4129 (3)	0.4340 (3)	0.0277 (7)	
H26	0.6671	0.4017	0.4960	0.033*	
C27	0.5933 (3)	0.4092 (3)	0.3618 (3)	0.0278 (8)	
H27	0.5143	0.3984	0.3744	0.033*	
C28	0.6209 (3)	0.4218 (3)	0.2718 (3)	0.0263 (8)	
H28	0.5610	0.4184	0.2204	0.032*	
C29	0.7366 (3)	0.4392 (3)	0.2567 (2)	0.0241 (7)	
C30	1.0440 (3)	0.4666 (3)	0.3872 (2)	0.0220 (7)	
C31	1.1492 (3)	0.4748 (3)	0.3470 (2)	0.0232 (7)	
H31	1.1472	0.4801	0.2809	0.028*	
C32	1.2568 (3)	0.4754 (3)	0.4023 (2)	0.0256 (7)	
H32	1.3273	0.4825	0.3734	0.031*	
C33	1.2639 (3)	0.4659 (3)	0.4994 (2)	0.0225 (7)	
C34	1.1585 (3)	0.4584 (3)	0.5390 (2)	0.0250 (7)	
H34	1.1610	0.4528	0.6051	0.030*	
C35	1.0491 (3)	0.4589 (3)	0.4846 (2)	0.0234 (7)	
H35	0.9784	0.4540	0.5136	0.028*	
C36	1.3811 (3)	0.4627 (3)	0.5588 (2)	0.0269 (7)	
H36A	1.3743	0.4966	0.6187	0.040*	
H36B	1.4405	0.4974	0.5261	0.040*	
H36C	1.4048	0.3920	0.5703	0.040*	
O7	0.2612 (2)	0.7100 (2)	0.10311 (17)	0.0298 (6)	
O8	0.0735 (2)	0.7151 (2)	0.05973 (17)	0.0349 (6)	
N10	0.3413 (3)	0.7031 (2)	0.2823 (2)	0.0218 (6)	
H10N	0.358 (3)	0.695 (3)	0.2239 (12)	0.026*	
N11	0.2005 (3)	0.7369 (3)	0.3827 (2)	0.0261 (7)	
N12	0.1578 (2)	0.7149 (2)	0.12156 (19)	0.0247 (6)	
C37	0.2271 (3)	0.7206 (3)	0.2953 (2)	0.0229 (7)	
C38	0.0884 (3)	0.7487 (3)	0.3965 (2)	0.0283 (8)	
H38	0.0712	0.7591	0.4590	0.034*	
C39	−0.0058 (3)	0.7470 (3)	0.3266 (3)	0.0300 (8)	
H39	−0.0846	0.7546	0.3411	0.036*	
C40	0.0185 (3)	0.7339 (3)	0.2358 (2)	0.0266 (7)	
H40	−0.0432	0.7328	0.1857	0.032*	
C41	0.1350 (3)	0.7223 (3)	0.2190 (2)	0.0239 (7)	
C42	0.4438 (3)	0.6970 (3)	0.3468 (2)	0.0223 (7)	
C43	0.5494 (3)	0.6836 (3)	0.3066 (2)	0.0243 (7)	
H43	0.5474	0.6785	0.2404	0.029*	
C44	0.6560 (3)	0.6777 (3)	0.3620 (2)	0.0252 (7)	
H44	0.7264	0.6688	0.3334	0.030*	
C45	0.6625 (3)	0.6846 (3)	0.4598 (3)	0.0238 (8)	
C46	0.5573 (3)	0.6970 (3)	0.4987 (2)	0.0239 (7)	
H46	0.5595	0.7013	0.5649	0.029*	
C47	0.4485 (3)	0.7033 (3)	0.4441 (2)	0.0259 (7)	
H47	0.3781	0.7117	0.4729	0.031*	
C48	0.7799 (3)	0.6812 (3)	0.5197 (3)	0.0293 (8)	
H48A	0.7675	0.6639	0.5842	0.044*	
H48B	0.8181	0.7477	0.5188	0.044*	
H48C	0.8303	0.6299	0.4950	0.044*	

### Atomic displacement parameters (Å^2^)

**Table:**

	*U*^11^	*U*^22^	*U*^33^	*U*^12^	*U*^13^	*U*^23^
O1	0.0286 (14)	0.0374 (15)	0.0249 (13)	0.0041 (11)	0.0009 (10)	−0.0004 (11)
O2	0.0310 (14)	0.0523 (18)	0.0343 (14)	0.0054 (13)	−0.0150 (11)	−0.0031 (13)
N1	0.0190 (14)	0.0333 (17)	0.0202 (14)	0.0008 (12)	−0.0008 (11)	−0.0027 (13)
N2	0.0210 (14)	0.0302 (17)	0.0265 (15)	0.0023 (12)	0.0003 (12)	−0.0023 (12)
N3	0.0255 (15)	0.0267 (16)	0.0289 (15)	0.0002 (12)	−0.0063 (12)	0.0009 (12)
C1	0.0182 (16)	0.0207 (17)	0.0256 (17)	−0.0017 (13)	−0.0016 (13)	0.0000 (13)
C2	0.0231 (17)	0.034 (2)	0.0352 (19)	−0.0009 (14)	0.0061 (14)	−0.0036 (15)
C3	0.0212 (17)	0.033 (2)	0.043 (2)	−0.0008 (15)	0.0045 (16)	0.0011 (16)
C4	0.0186 (16)	0.0265 (19)	0.0375 (19)	−0.0030 (13)	−0.0052 (14)	0.0069 (15)
C5	0.0219 (16)	0.0230 (18)	0.0275 (17)	−0.0004 (13)	−0.0004 (13)	0.0012 (13)
C6	0.0162 (16)	0.0233 (17)	0.0236 (17)	−0.0003 (12)	−0.0033 (13)	−0.0003 (13)
C7	0.0256 (17)	0.0284 (19)	0.0208 (16)	−0.0031 (14)	0.0021 (13)	−0.0014 (13)
C8	0.0170 (15)	0.0293 (19)	0.0273 (18)	−0.0010 (13)	0.0034 (13)	−0.0029 (14)
C9	0.0205 (16)	0.0212 (17)	0.0297 (19)	0.0008 (13)	−0.0018 (14)	−0.0008 (14)
C10	0.0255 (18)	0.0260 (18)	0.0207 (16)	0.0010 (14)	−0.0010 (13)	0.0008 (13)
C11	0.0201 (16)	0.0277 (18)	0.0231 (16)	0.0004 (13)	0.0027 (13)	0.0019 (14)
C12	0.0249 (17)	0.0316 (19)	0.0267 (18)	0.0011 (14)	−0.0039 (14)	0.0014 (14)
O3	0.0304 (14)	0.0431 (16)	0.0297 (13)	−0.0026 (12)	−0.0052 (11)	0.0014 (11)
O4	0.0379 (16)	0.0442 (18)	0.0375 (16)	−0.0008 (13)	−0.0194 (13)	−0.0036 (13)
N4	0.0200 (14)	0.0316 (17)	0.0215 (14)	−0.0030 (12)	−0.0008 (11)	0.0005 (12)
N5	0.0216 (15)	0.0338 (18)	0.0345 (17)	0.0002 (13)	0.0018 (12)	0.0002 (14)
N6	0.0310 (17)	0.0281 (16)	0.0289 (16)	0.0009 (13)	−0.0089 (13)	−0.0034 (13)
C13	0.0202 (17)	0.0234 (19)	0.0308 (19)	0.0044 (14)	−0.0022 (14)	−0.0033 (15)
C14	0.0247 (18)	0.037 (2)	0.037 (2)	−0.0022 (15)	0.0057 (15)	−0.0036 (16)
C15	0.0179 (18)	0.033 (2)	0.057 (3)	0.0013 (15)	0.0061 (17)	−0.0118 (18)
C16	0.0225 (19)	0.030 (2)	0.044 (2)	0.0050 (15)	−0.0055 (16)	−0.0085 (17)
C17	0.0220 (17)	0.0215 (18)	0.034 (2)	0.0033 (14)	−0.0048 (15)	−0.0064 (14)
C18	0.0202 (17)	0.0210 (17)	0.0251 (18)	−0.0008 (13)	−0.0002 (14)	0.0010 (13)
C19	0.0218 (16)	0.0268 (18)	0.0199 (15)	−0.0026 (13)	0.0013 (13)	−0.0005 (13)
C20	0.0185 (16)	0.0264 (17)	0.0277 (17)	0.0019 (13)	0.0040 (13)	−0.0004 (14)
C21	0.0205 (16)	0.0223 (17)	0.0250 (17)	−0.0011 (13)	−0.0022 (13)	0.0025 (13)
C22	0.0234 (17)	0.0279 (18)	0.0193 (16)	−0.0028 (14)	0.0002 (13)	−0.0010 (13)
C23	0.0175 (16)	0.0270 (18)	0.0252 (17)	−0.0009 (13)	0.0015 (13)	−0.0022 (13)
C24	0.0217 (17)	0.0321 (19)	0.0257 (17)	0.0004 (14)	−0.0018 (14)	0.0022 (14)
O5	0.0248 (13)	0.0582 (19)	0.0236 (12)	−0.0023 (12)	0.0006 (10)	0.0065 (12)
O6	0.0304 (14)	0.0401 (16)	0.0245 (13)	0.0015 (12)	−0.0096 (10)	0.0010 (11)
N7	0.0208 (14)	0.0342 (17)	0.0192 (14)	−0.0009 (12)	−0.0014 (11)	0.0030 (12)
N8	0.0190 (14)	0.0295 (16)	0.0235 (14)	−0.0007 (12)	0.0003 (11)	−0.0001 (12)
N9	0.0246 (14)	0.0298 (16)	0.0238 (14)	0.0025 (12)	−0.0048 (11)	0.0020 (12)
C25	0.0203 (16)	0.0202 (17)	0.0237 (17)	0.0014 (13)	−0.0016 (13)	0.0008 (13)
C26	0.0215 (16)	0.0310 (19)	0.0313 (18)	−0.0006 (14)	0.0056 (14)	−0.0013 (15)
C27	0.0164 (16)	0.033 (2)	0.0334 (19)	0.0003 (14)	0.0017 (14)	−0.0011 (15)
C28	0.0194 (17)	0.0278 (19)	0.0299 (19)	0.0021 (14)	−0.0050 (14)	−0.0026 (15)
C29	0.0250 (17)	0.0234 (18)	0.0232 (17)	0.0018 (14)	−0.0002 (14)	0.0017 (14)
C30	0.0202 (16)	0.0230 (18)	0.0219 (17)	−0.0002 (13)	−0.0017 (13)	−0.0018 (13)
C31	0.0223 (16)	0.0275 (18)	0.0197 (16)	−0.0022 (13)	0.0015 (13)	0.0026 (13)
C32	0.0189 (16)	0.0282 (19)	0.0301 (18)	−0.0020 (13)	0.0044 (13)	0.0011 (14)
C33	0.0180 (16)	0.0213 (17)	0.0271 (17)	−0.0005 (13)	−0.0023 (13)	−0.0024 (13)
C34	0.0219 (16)	0.0287 (19)	0.0234 (17)	−0.0007 (14)	−0.0016 (13)	−0.0015 (13)
C35	0.0166 (15)	0.0294 (19)	0.0235 (16)	−0.0003 (13)	−0.0005 (12)	−0.0030 (13)
C36	0.0202 (16)	0.0283 (19)	0.0308 (18)	−0.0004 (14)	−0.0029 (14)	−0.0030 (14)
O7	0.0229 (13)	0.0441 (16)	0.0223 (12)	0.0019 (11)	0.0013 (10)	−0.0010 (11)
O8	0.0280 (14)	0.0488 (17)	0.0250 (12)	0.0057 (12)	−0.0100 (10)	−0.0051 (12)
N10	0.0183 (14)	0.0281 (16)	0.0188 (14)	0.0020 (12)	0.0015 (11)	−0.0013 (12)
N11	0.0232 (15)	0.0319 (18)	0.0234 (15)	0.0020 (12)	0.0031 (12)	0.0040 (12)
N12	0.0223 (14)	0.0296 (16)	0.0205 (14)	0.0038 (12)	−0.0053 (11)	0.0000 (12)
C37	0.0210 (17)	0.0237 (18)	0.0235 (16)	−0.0001 (14)	0.0001 (13)	−0.0014 (14)
C38	0.0227 (17)	0.040 (2)	0.0230 (17)	0.0004 (14)	0.0057 (13)	0.0000 (15)
C39	0.0203 (17)	0.035 (2)	0.035 (2)	−0.0006 (14)	0.0059 (15)	0.0015 (15)
C40	0.0197 (17)	0.0306 (19)	0.0277 (17)	−0.0011 (14)	−0.0049 (13)	−0.0017 (14)
C41	0.0221 (16)	0.0254 (18)	0.0239 (17)	−0.0006 (13)	0.0006 (13)	0.0004 (13)
C42	0.0185 (17)	0.0231 (18)	0.0247 (17)	−0.0008 (13)	−0.0006 (13)	0.0002 (13)
C43	0.0214 (17)	0.0276 (19)	0.0239 (17)	0.0012 (13)	0.0020 (14)	0.0002 (13)
C44	0.0209 (17)	0.0276 (18)	0.0270 (17)	0.0004 (13)	0.0024 (13)	−0.0002 (13)
C45	0.0213 (17)	0.0199 (17)	0.0288 (19)	0.0007 (12)	−0.0032 (15)	0.0015 (14)
C46	0.0228 (17)	0.0277 (18)	0.0199 (16)	−0.0010 (13)	−0.0030 (13)	0.0021 (13)
C47	0.0242 (17)	0.0286 (19)	0.0245 (17)	−0.0020 (15)	0.0011 (14)	0.0002 (14)
C48	0.0230 (18)	0.031 (2)	0.0315 (19)	−0.0004 (14)	−0.0068 (15)	0.0011 (15)

### Geometric parameters (Å, º)

**Table:**

O1—N3	1.249 (4)	O5—N9	1.241 (4)
O2—N3	1.239 (4)	O6—N9	1.239 (4)
N1—C1	1.345 (4)	N7—C25	1.351 (4)
N1—C6	1.416 (4)	N7—C30	1.411 (4)
N1—H1N	0.882 (10)	N7—H7N	0.880 (10)
N2—C2	1.322 (5)	N8—C26	1.339 (4)
N2—C1	1.353 (5)	N8—C25	1.353 (4)
N3—C5	1.442 (4)	N9—C29	1.450 (4)
C1—C5	1.435 (5)	C25—C29	1.433 (5)
C2—C3	1.393 (5)	C26—C27	1.394 (5)
C2—H2	0.9500	C26—H26	0.9500
C3—C4	1.374 (5)	C27—C28	1.375 (5)
C3—H3	0.9500	C27—H27	0.9500
C4—C5	1.392 (4)	C28—C29	1.380 (5)
C4—H4	0.9500	C28—H28	0.9500
C6—C11	1.393 (5)	C30—C31	1.393 (4)
C6—C7	1.400 (5)	C30—C35	1.397 (5)
C7—C8	1.384 (5)	C31—C32	1.386 (5)
C7—H7	0.9500	C31—H31	0.9500
C8—C9	1.397 (5)	C32—C33	1.393 (5)
C8—H8	0.9500	C32—H32	0.9500
C9—C10	1.391 (5)	C33—C34	1.390 (4)
C9—C12	1.510 (4)	C33—C36	1.505 (4)
C10—C11	1.392 (5)	C34—C35	1.398 (4)
C10—H10	0.9500	C34—H34	0.9500
C11—H11	0.9500	C35—H35	0.9500
C12—H12A	0.9800	C36—H36A	0.9800
C12—H12B	0.9800	C36—H36B	0.9800
C12—H12C	0.9800	C36—H36C	0.9800
O3—N6	1.245 (4)	O7—N12	1.240 (4)
O4—N6	1.238 (4)	O8—N12	1.234 (4)
N4—C13	1.347 (5)	N10—C37	1.356 (4)
N4—C18	1.421 (4)	N10—C42	1.412 (4)
N4—H4N	0.880 (10)	N10—H10N	0.888 (10)
N5—C14	1.331 (5)	N11—C38	1.325 (4)
N5—C13	1.349 (5)	N11—C37	1.343 (4)
N6—C17	1.445 (5)	N12—C41	1.455 (4)
C13—C17	1.434 (5)	C37—C41	1.432 (5)
C14—C15	1.394 (6)	C38—C39	1.387 (5)
C14—H14	0.9500	C38—H38	0.9500
C15—C16	1.368 (6)	C39—C40	1.375 (5)
C15—H15	0.9500	C39—H39	0.9500
C16—C17	1.385 (5)	C40—C41	1.385 (5)
C16—H16	0.9500	C40—H40	0.9500
C18—C19	1.390 (5)	C42—C47	1.395 (5)
C18—C23	1.396 (5)	C42—C43	1.405 (5)
C19—C20	1.383 (5)	C43—C44	1.380 (5)
C19—H19	0.9500	C43—H43	0.9500
C20—C21	1.395 (5)	C44—C45	1.402 (5)
C20—H20	0.9500	C44—H44	0.9500
C21—C22	1.390 (5)	C45—C46	1.388 (5)
C21—C24	1.503 (4)	C45—C48	1.510 (4)
C22—C23	1.391 (5)	C46—C47	1.396 (5)
C22—H22	0.9500	C46—H46	0.9500
C23—H23	0.9500	C47—H47	0.9500
C24—H24A	0.9800	C48—H48A	0.9800
C24—H24B	0.9800	C48—H48B	0.9800
C24—H24C	0.9800	C48—H48C	0.9800
			
C1—N1—C6	130.5 (3)	C25—N7—C30	132.1 (3)
C1—N1—H1N	115 (3)	C25—N7—H7N	114 (3)
C6—N1—H1N	114 (3)	C30—N7—H7N	114 (3)
C2—N2—C1	119.0 (3)	C26—N8—C25	118.5 (3)
O2—N3—O1	121.7 (3)	O6—N9—O5	122.0 (3)
O2—N3—C5	119.0 (3)	O6—N9—C29	118.6 (3)
O1—N3—C5	119.3 (3)	O5—N9—C29	119.5 (3)
N1—C1—N2	118.1 (3)	N7—C25—N8	118.0 (3)
N1—C1—C5	122.8 (3)	N7—C25—C29	122.6 (3)
N2—C1—C5	119.0 (3)	N8—C25—C29	119.4 (3)
N2—C2—C3	125.4 (4)	N8—C26—C27	124.7 (3)
N2—C2—H2	117.3	N8—C26—H26	117.7
C3—C2—H2	117.3	C27—C26—H26	117.7
C4—C3—C2	117.2 (3)	C28—C27—C26	117.6 (3)
C4—C3—H3	121.4	C28—C27—H27	121.2
C2—C3—H3	121.4	C26—C27—H27	121.2
C3—C4—C5	119.3 (3)	C27—C28—C29	119.4 (3)
C3—C4—H4	120.3	C27—C28—H28	120.3
C5—C4—H4	120.3	C29—C28—H28	120.3
C4—C5—C1	120.1 (3)	C28—C29—C25	120.3 (3)
C4—C5—N3	117.4 (3)	C28—C29—N9	117.6 (3)
C1—C5—N3	122.4 (3)	C25—C29—N9	122.0 (3)
C11—C6—C7	119.1 (3)	C31—C30—C35	118.6 (3)
C11—C6—N1	125.2 (3)	C31—C30—N7	115.3 (3)
C7—C6—N1	115.6 (3)	C35—C30—N7	126.1 (3)
C8—C7—C6	120.3 (3)	C32—C31—C30	120.8 (3)
C8—C7—H7	119.9	C32—C31—H31	119.6
C6—C7—H7	119.9	C30—C31—H31	119.6
C7—C8—C9	121.4 (3)	C31—C32—C33	121.5 (3)
C7—C8—H8	119.3	C31—C32—H32	119.3
C9—C8—H8	119.3	C33—C32—H32	119.3
C10—C9—C8	117.6 (3)	C34—C33—C32	117.4 (3)
C10—C9—C12	121.3 (3)	C34—C33—C36	121.3 (3)
C8—C9—C12	121.1 (3)	C32—C33—C36	121.3 (3)
C9—C10—C11	122.0 (3)	C33—C34—C35	122.0 (3)
C9—C10—H10	119.0	C33—C34—H34	119.0
C11—C10—H10	119.0	C35—C34—H34	119.0
C10—C11—C6	119.7 (3)	C30—C35—C34	119.7 (3)
C10—C11—H11	120.2	C30—C35—H35	120.2
C6—C11—H11	120.2	C34—C35—H35	120.2
C9—C12—H12A	109.5	C33—C36—H36A	109.5
C9—C12—H12B	109.5	C33—C36—H36B	109.5
H12A—C12—H12B	109.5	H36A—C36—H36B	109.5
C9—C12—H12C	109.5	C33—C36—H36C	109.5
H12A—C12—H12C	109.5	H36A—C36—H36C	109.5
H12B—C12—H12C	109.5	H36B—C36—H36C	109.5
C13—N4—C18	130.5 (3)	C37—N10—C42	131.1 (3)
C13—N4—H4N	113 (3)	C37—N10—H10N	118 (3)
C18—N4—H4N	115 (3)	C42—N10—H10N	111 (3)
C14—N5—C13	119.1 (3)	C38—N11—C37	118.8 (3)
O4—N6—O3	121.8 (3)	O8—N12—O7	122.0 (3)
O4—N6—C17	118.5 (3)	O8—N12—C41	119.0 (3)
O3—N6—C17	119.7 (3)	O7—N12—C41	119.0 (3)
N4—C13—N5	118.2 (3)	N11—C37—N10	118.5 (3)
N4—C13—C17	122.4 (3)	N11—C37—C41	119.4 (3)
N5—C13—C17	119.4 (3)	N10—C37—C41	122.1 (3)
N5—C14—C15	124.3 (4)	N11—C38—C39	124.9 (3)
N5—C14—H14	117.9	N11—C38—H38	117.5
C15—C14—H14	117.9	C39—C38—H38	117.5
C16—C15—C14	117.9 (4)	C40—C39—C38	117.9 (3)
C16—C15—H15	121.1	C40—C39—H39	121.1
C14—C15—H15	121.1	C38—C39—H39	121.1
C15—C16—C17	119.4 (4)	C39—C40—C41	118.6 (3)
C15—C16—H16	120.3	C39—C40—H40	120.7
C17—C16—H16	120.3	C41—C40—H40	120.7
C16—C17—C13	119.9 (4)	C40—C41—C37	120.3 (3)
C16—C17—N6	117.6 (3)	C40—C41—N12	116.9 (3)
C13—C17—N6	122.4 (3)	C37—C41—N12	122.7 (3)
C19—C18—C23	119.6 (3)	C47—C42—C43	118.8 (3)
C19—C18—N4	115.8 (3)	C47—C42—N10	126.3 (3)
C23—C18—N4	124.6 (3)	C43—C42—N10	115.0 (3)
C20—C19—C18	120.0 (3)	C44—C43—C42	120.7 (3)
C20—C19—H19	120.0	C44—C43—H43	119.7
C18—C19—H19	120.0	C42—C43—H43	119.7
C19—C20—C21	121.8 (3)	C43—C44—C45	121.3 (3)
C19—C20—H20	119.1	C43—C44—H44	119.3
C21—C20—H20	119.1	C45—C44—H44	119.3
C22—C21—C20	117.2 (3)	C46—C45—C44	117.3 (3)
C22—C21—C24	121.4 (3)	C46—C45—C48	121.7 (3)
C20—C21—C24	121.4 (3)	C44—C45—C48	120.9 (3)
C21—C22—C23	122.3 (3)	C45—C46—C47	122.4 (3)
C21—C22—H22	118.9	C45—C46—H46	118.8
C23—C22—H22	118.9	C47—C46—H46	118.8
C22—C23—C18	119.1 (3)	C46—C47—C42	119.5 (3)
C22—C23—H23	120.4	C46—C47—H47	120.2
C18—C23—H23	120.4	C42—C47—H47	120.2
C21—C24—H24A	109.5	C45—C48—H48A	109.5
C21—C24—H24B	109.5	C45—C48—H48B	109.5
H24A—C24—H24B	109.5	H48A—C48—H48B	109.5
C21—C24—H24C	109.5	C45—C48—H48C	109.5
H24A—C24—H24C	109.5	H48A—C48—H48C	109.5
H24B—C24—H24C	109.5	H48B—C48—H48C	109.5
			
C6—N1—C1—N2	0.1 (6)	C30—N7—C25—N8	6.6 (6)
C6—N1—C1—C5	−179.8 (3)	C30—N7—C25—C29	−173.1 (3)
C2—N2—C1—N1	−179.9 (3)	C26—N8—C25—N7	178.8 (3)
C2—N2—C1—C5	−0.1 (5)	C26—N8—C25—C29	−1.4 (5)
C1—N2—C2—C3	−0.2 (6)	C25—N8—C26—C27	−1.0 (6)
N2—C2—C3—C4	0.2 (6)	N8—C26—C27—C28	2.3 (6)
C2—C3—C4—C5	0.2 (6)	C26—C27—C28—C29	−1.3 (6)
C3—C4—C5—C1	−0.5 (6)	C27—C28—C29—C25	−1.0 (6)
C3—C4—C5—N3	176.5 (3)	C27—C28—C29—N9	177.6 (4)
N1—C1—C5—C4	−179.7 (3)	N7—C25—C29—C28	−177.9 (4)
N2—C1—C5—C4	0.4 (5)	N8—C25—C29—C28	2.4 (6)
N1—C1—C5—N3	3.5 (6)	N7—C25—C29—N9	3.7 (6)
N2—C1—C5—N3	−176.4 (3)	N8—C25—C29—N9	−176.1 (3)
O2—N3—C5—C4	4.3 (5)	O6—N9—C29—C28	0.0 (5)
O1—N3—C5—C4	−175.0 (3)	O5—N9—C29—C28	−178.8 (4)
O2—N3—C5—C1	−178.8 (3)	O6—N9—C29—C25	178.5 (3)
O1—N3—C5—C1	1.9 (5)	O5—N9—C29—C25	−0.3 (5)
C1—N1—C6—C11	−25.8 (7)	C25—N7—C30—C31	169.7 (4)
C1—N1—C6—C7	157.6 (4)	C25—N7—C30—C35	−9.7 (6)
C11—C6—C7—C8	0.6 (6)	C35—C30—C31—C32	0.0 (5)
N1—C6—C7—C8	177.5 (3)	N7—C30—C31—C32	−179.5 (3)
C6—C7—C8—C9	−0.2 (6)	C30—C31—C32—C33	1.3 (6)
C7—C8—C9—C10	−0.1 (5)	C31—C32—C33—C34	−1.7 (5)
C7—C8—C9—C12	179.0 (3)	C31—C32—C33—C36	177.7 (3)
C8—C9—C10—C11	0.0 (5)	C32—C33—C34—C35	0.9 (5)
C12—C9—C10—C11	−179.2 (3)	C36—C33—C34—C35	−178.5 (3)
C9—C10—C11—C6	0.5 (6)	C31—C30—C35—C34	−0.8 (5)
C7—C6—C11—C10	−0.8 (5)	N7—C30—C35—C34	178.7 (3)
N1—C6—C11—C10	−177.3 (3)	C33—C34—C35—C30	0.3 (5)
C18—N4—C13—N5	−2.2 (6)	C38—N11—C37—N10	−176.4 (4)
C18—N4—C13—C17	176.0 (3)	C38—N11—C37—C41	3.6 (6)
C14—N5—C13—N4	179.0 (3)	C42—N10—C37—N11	0.9 (6)
C14—N5—C13—C17	0.7 (6)	C42—N10—C37—C41	−179.1 (4)
C13—N5—C14—C15	−0.2 (6)	C37—N11—C38—C39	−1.0 (6)
N5—C14—C15—C16	0.5 (6)	N11—C38—C39—C40	−1.2 (6)
C14—C15—C16—C17	−1.4 (6)	C38—C39—C40—C41	0.6 (6)
C15—C16—C17—C13	1.9 (6)	C39—C40—C41—C37	2.1 (6)
C15—C16—C17—N6	−176.8 (4)	C39—C40—C41—N12	−176.3 (3)
N4—C13—C17—C16	−179.8 (3)	N11—C37—C41—C40	−4.2 (6)
N5—C13—C17—C16	−1.6 (6)	N10—C37—C41—C40	175.8 (3)
N4—C13—C17—N6	−1.2 (6)	N11—C37—C41—N12	174.0 (3)
N5—C13—C17—N6	177.0 (3)	N10—C37—C41—N12	−6.0 (6)
O4—N6—C17—C16	0.6 (5)	O8—N12—C41—C40	−2.9 (5)
O3—N6—C17—C16	179.9 (3)	O7—N12—C41—C40	176.1 (3)
O4—N6—C17—C13	−178.0 (3)	O8—N12—C41—C37	178.8 (3)
O3—N6—C17—C13	1.3 (5)	O7—N12—C41—C37	−2.1 (5)
C13—N4—C18—C19	−153.3 (4)	C37—N10—C42—C47	3.7 (7)
C13—N4—C18—C23	29.0 (6)	C37—N10—C42—C43	−176.3 (4)
C23—C18—C19—C20	−0.3 (6)	C47—C42—C43—C44	−0.7 (6)
N4—C18—C19—C20	−178.1 (3)	N10—C42—C43—C44	179.3 (3)
C18—C19—C20—C21	−1.0 (6)	C42—C43—C44—C45	0.2 (6)
C19—C20—C21—C22	1.5 (5)	C43—C44—C45—C46	0.4 (5)
C19—C20—C21—C24	−177.6 (3)	C43—C44—C45—C48	−178.2 (3)
C20—C21—C22—C23	−0.6 (5)	C44—C45—C46—C47	−0.5 (5)
C24—C21—C22—C23	178.5 (3)	C48—C45—C46—C47	178.0 (3)
C21—C22—C23—C18	−0.6 (5)	C45—C46—C47—C42	0.0 (6)
C19—C18—C23—C22	1.1 (5)	C43—C42—C47—C46	0.6 (5)
N4—C18—C23—C22	178.7 (3)	N10—C42—C47—C46	−179.3 (4)

### Hydrogen-bond geometry (Å, º)

**Table:**

*D*—H···*A*	*D*—H	H···*A*	*D*···*A*	*D*—H···*A*
N1—H1*N*···O1	0.88 (2)	1.93 (2)	2.632 (4)	135 (3)
N4—H4*N*···O3	0.88 (2)	1.92 (3)	2.630 (4)	137 (4)
N7—H7*N*···O5	0.88 (2)	1.92 (3)	2.622 (4)	136 (4)
N10—H10*N*···O7	0.89 (2)	1.96 (2)	2.636 (4)	132 (3)
C31—H31···O1^i^	0.95	2.50	3.444 (4)	173
C28—H28···O2^ii^	0.95	2.59	3.414 (4)	145
C4—H4···O6^iii^	0.95	2.55	3.440 (4)	157
C7—H7···O5^iv^	0.95	2.38	3.331 (4)	174
C43—H43···O3^ii^	0.95	2.49	3.436 (4)	172
C40—H40···O4^v^	0.95	2.64	3.489 (5)	149
C19—H19···O7^iii^	0.95	2.42	3.364 (4)	176
C16—H16···O8^vi^	0.95	2.52	3.398 (4)	153
N3—O3···*Cg*(N5,C13–C17)^vii^	1.24 (1)	3.55 (1)	3.449 (3)	75 (1)
N6—O4···*Cg*(N2,C1–C5)^viii^	1.24 (1)	3.46 (1)	3.469 (3)	80 (1)
C36—H36*C*···*Cg*(C42–C47)^ix^	0.98	2.72	3.698 (4)	174
C48—H48*B*···*Cg*(C30–C35)^x^	0.98	2.95	3.868 (4)	156

Symmetry codes: (i) *x*+1, *y*, *z*−1; (ii) *x*, *y*, *z*−1; (iii) *x*, *y*, *z*+1; (iv) *x*−1, *y*, *z*+1; (v) *x*−1, *y*, *z*−1; (vi) *x*+1, *y*, *z*+1; (vii) −*x*+1, *y*−1/2, −*z*+2; (viii) −*x*+1, *y*+1/2, −*z*+2; (ix) −*x*+2, *y*−1/2, −*z*+1; (x) −*x*+2, *y*+1/2, −*z*+1.

## References

[bb1] Abdullah, Z. (2005). *Int. J. Chem. Sci.* **3**, 9–15.

[bb2] Agilent (2013). *CrysAlis PRO* Agilent Technologies Inc., Santa Clara, CA, USA.

[bb3] Akhmad Aznan, A. M., Fairuz, Z. A., Abdullah, Z., Ng, S. W. & Tiekink, E. R. T. (2011). *Acta Cryst.* E**67**, o3176. 10.1107/S1600536811045570 PMC3238847 22199700

[bb4] Arman, H. D., Kaulgud, T., Miller, T. & Tiekink, E. R. T. (2014). *Z. Kristallogr.* **229**, 295–302.

[bb5] Arman, H. D. & Tiekink, E. R. T. (2013). *Z. Kristallogr.* **228**, 289–294.

[bb6] Arora, K. K. & Zaworotko, M. J. (2009). *Polymorphism in Pharmaceutical Solids*, Vol. 2, edited by H. G. Brittain, p. 281. London: Informa Healthcare.

[bb7] Aznan Akhmad, M. A., Abdullah, Z., Fairuz, Z. A., Ng, S. W. & Tiekink, E. R. T. (2010). *Acta Cryst.* E**66**, o2400. 10.1107/S1600536810033040 PMC3008084 21588732

[bb8] Brandenburg, K. (2006). *DIAMOND* Crystal Impact GbR, Bonn, Germany.

[bb9] Cao, S.-L., Zhao, J., Zhang, N., Wang, Y., Jiang, Y.-Y. & Feng, Y.-P. (2011). *J. Chem. Crystallogr.* **41**, 1456–1460.

[bb10] Farrugia, L. J. (2012). *J. Appl. Cryst.* **45**, 849–854.

[bb11] Frisch, M. J., *et al.* (2009). *GAUSSIAN09* Gaussian Inc., Wallingford, CT, USA.

[bb12] Gans, J. & Shalloway, D. (2001). *J. Mol. Graph. Model.* **19**, 557–559. 10.1016/s1093-3263(01)00090-0 11552684

[bb13] Kawai, M., Lee, M. J., Evans, K. O. & Norlund, T. (2001). *J. Fluoresc.* **11**, 23–32.

[bb14] McWilliam, S. A., Skakle, J. M. S., Wardell, J. L., Low, J. N. & Glidewell, C. (2001). *Acta Cryst.* C**57**, 946–948. 10.1107/s0108270101008010 11498622

[bb15] Shattock, T., Arora, K. K., Vishweshwar, P. & Zaworotko, M. J. (2008). *Cryst. Growth Des.* **8**, 4533–4545.

[bb16] Sheldrick, G. M. (2008). *Acta Cryst.* A**64**, 112–122. 10.1107/S0108767307043930 18156677

[bb17] Spek, A. L. (2009). *Acta Cryst.* D**65**, 148–155. 10.1107/S090744490804362X PMC2631630 19171970

[bb18] Vishweshwar, P., McMahon, J. A., Oliveira, M., Peterson, M. L. & Zaworotko, M. J. (2005). *J. Am. Chem. Soc.* **127**, 16802–16803. 10.1021/ja056455b 16316223

[bb19] Westrip, S. P. (2010). *J. Appl. Cryst.* **43**, 920–925.

